# Branched-Chain Amino Acid Assembly into Amyloid-like Fibrils Provides a New Paradigm for Maple Syrup Urine Disease Pathology

**DOI:** 10.3390/ijms242115999

**Published:** 2023-11-06

**Authors:** Topaz Kreiser, Ilana Sogolovsky-Bard, Dor Zaguri, Shira Shaham-Niv, Dana Laor Bar-Yosef, Ehud Gazit

**Affiliations:** 1 The Shmunis School of Biomedicine and Cancer Research, Tel Aviv University, Tel Aviv 6997801, Israel; topazk136@gmail.com (T.K.); ilanasogo@tauex.tau.ac.il (I.S.-B.); dorszaguri@gmail.com (D.Z.); shira.shaham@gmail.com (S.S.-N.); danalaor@tauex.tau.ac.il (D.L.B.-Y.); 2 Department of Cell and Developmental Biology, Sackler School of Medicine, Tel Aviv University, Tel Aviv 6997801, Israel; 3 Blavatnik Center for Drug Discovery, Tel Aviv University, Tel Aviv 6997801, Israel; 4 Sagol School of Neuroscience, Tel Aviv University, Tel Aviv 6997801, Israel

**Keywords:** BCAAs, self-assembly, amyloid-like structures, metabolite amyloids, metabolostasis, MSUD pathology, polyphenols

## Abstract

Inborn error of metabolism disorders (IEMs) are a family of diseases resulting from single-gene mutations that lead to the accumulation of metabolites that are usually toxic or interfere with normal cell function. The etiological link between metabolic alteration and the symptoms of IEMs is still elusive. Several metabolites, which accumulate in IEMs, were shown to self-assemble to form ordered structures. These structures display the same biophysical, biochemical, and biological characteristics as proteinaceous amyloid fibrils. Here, we have demonstrated, for the first time, the ability of each of the branched-chain amino acids (BCAAs) that accumulate in maple syrup urine disease (MSUD) to self-assemble into amyloid-like fibrils depicted by characteristic morphology, binding to indicative amyloid-specific dyes and dose-dependent cytotoxicity by a late apoptosis mechanism. We could also detect the presence of the assemblies in living cells. In addition, by employing several in vitro techniques, we demonstrated the ability of known polyphenols to inhibit the formation of the BCAA fibrils. Our study implies that BCAAs possess a pathological role in MSUD, extends the paradigm-shifting concept regarding the toxicity of metabolite amyloid-like structures, and suggests new pathological targets that may lead to highly needed novel therapeutic opportunities for this orphan disease.

## 1. Introduction

Rare diseases affect more than 300 million patients worldwide [1]. Inborn errors of metabolism disorders (IEMs) are a group of over 1000 heterogeneous disorders, and although most disorders are orphan diseases, IEMs are collectively frequent [2,3]. Maple syrup urine disease (MSUD) is a rare, autosomal recessive IEM disorder that affects the catabolism of branched-chain amino acids (BCAAs), leucine, isoleucine, and valine. MSUD equally affects both men and women with an estimated incidence of approximately 1 case per 180,000 live births globally. It classically manifests in the neonatal period with failure to thrive and, if left untreated, can result in various complications including poor feeding, vomiting, lethargy, developmental delays, seizures, coma, or death in severe cases [4,5].

The disorder is caused by mutations in the genes that code for the enzyme complex responsible for the breakdown of BCAAs. As a result, individuals with MSUD accumulate high levels of BCAAs in blood and tissues, which can cause a range of neurological symptoms. The high levels of these amino acids result in the appearance of a substance named sotolone [6], which leads to a maple syrup odor in the urine and cerumen [5,7].

MSUD has no disease-modifying drug therapy, with treatment consisting of close metabolic monitoring and the avoidance of the consumption of accumulating BCAAs by employing an extraordinarily strict diet [8]. Liver transplantation is an alternative approach to significantly improve outcomes. However, this invasive operation presents its own challenges to the patients [9,10]. As of today, only one clinical trial concerning MSUD therapy has been conducted; therefore, new therapeutic approaches such as efficacious small molecules are desperately needed [11,12].

Despite being first described almost 70 years ago [13], the precise etiological mechanisms underlying MSUD pathogenesis remain poorly understood. While it is known that high levels of BCAAs result in a compromised energy metabolism and a diminished rate of protein synthesis [14], the precise molecular and cellular processes that drive the pathophysiology of MSUD have yet to be fully elucidated, resulting in no remedy for the patients [15,16]. Recently, several metabolites associated with various IEMs, including both single amino acids and nucleobases, were demonstrated to self-assemble and form amyloid-like fibrillar assemblies. These findings support the metabolite amyloid hypothesis, suggesting that small, monomeric metabolites can self-assemble without external factors to form amyloid-like fibrils showing similar properties to proteinaceous amyloids [17,18,19,20]. This has led us to surmise whether the self-assembly and aggregation of BCAAs occur and whether they contribute to MSUD pathogenesis.

BCAAs are hydrophobic amino acids that tend to cluster together in solution due to favorable intermolecular interactions [21,22]. Under normal physiological conditions, BCAAs are rapidly metabolized by the mitochondrial enzyme complex branched-chain alpha-keto acid dehydrogenase (BCKDH), which catalyzes the oxidative decarboxylation of BCAAs to their corresponding alpha-keto acids. However, in individuals with MSUD, mutations in the BCKDH complex result in impaired enzymatic activity and the accumulation of BCAAs [5,23].

Here, we present novel evidence that supports the hypothesis that elevated levels of BCAAs trigger the self-assembly and subsequent aggregation of these amino acids, resulting in the formation of amyloid-like structures. These assemblies exhibit several hallmarks of amyloid fibrils including a characteristic fibrillar morphology, binding to amyloid-specific dyes, and a dose-dependent cytotoxic effect. The assemblies can also be detected in living cells using an amyloid-specific dye that is able to penetrate the cell membrane. In addition, employing several in vitro techniques, we demonstrated the inhibition of the BCAA structures by known polyphenols that target amyloid formation.

Overall, our findings suggest an innovative mechanism for MSUD pathology, involving the self-assembly of BCAAs, which results in the formation of amyloid-like structures. Moreover, our results show that targeting the aggregation of BCAAs with amyloid inhibitors can rescue these effects. Our study provides a new perspective on the pathogenesis of MSUD and highlights the potential of amyloid inhibitors as a therapeutic strategy for this devastating disease. Further research is needed to fully understand the mechanisms underlying BCAA aggregation and its effects on cellular and physiological processes, as well as to develop safe and effective small molecule inhibitors for clinical use in MSUD.

## 2. Results

### 2.1. Analysis of Structure Formation by Branched-Chain Amino Acids

To examine our hypothesis that high levels of BCAAs prompt their self-assembly, we aimed to study their ability to form ordered structures. In an attempt to mimic physiological pH and ionic strength, we used phosphate-buffered saline (PBS) as the medium to study the possible molecular interactions.

**Figure 1:**
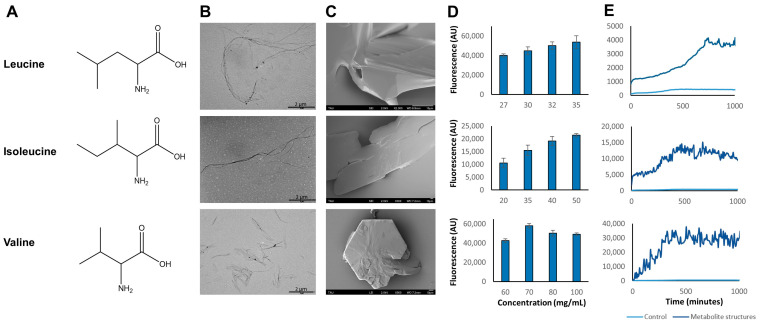
Formation of structures by BCAA self-assembly. (**A**) Skeletal formula of leucine, isoleucine, and valine. (**B**,**C**) An amount of 4 mg/mL of each of the metabolites was dissolved at 90 °C to ascertain the monomeric state and then cooled down gradually. (**B**) Transmission electron microscope (TEM) micrographs were obtained 24 h after inducing self-assembly. Scale bars: 2 µm. (**C**) High-resolution scanning electron microscope (HR-SEM) micrographs showing the metabolite crystal structures. Scale bars: 10 µm. (**D**,**E**) Observation of branched-chain amino acids (BCAA) assemblies by amyloid-specific fluorescent dyes. Metabolites were dissolved at 90 °C in phosphate-buffered saline (PBS), followed by the addition of amyloid-specific fluorescent dyes. The control represents PBS alone without the addition of metabolites. (**D**) Endpoint measurements of fluorescence signals obtained following the addition of ProteoStat^®^ (emission signal at 620 nm, excitation at 485 nm) to structure solutions of the BCAAs. (**E**) The 40 μM Thioflavin-T (ThT) emission data at 480 nm (excitation at 440 nm), presenting the kinetics of the structure formation (light blue) of leucine (35 mg/mL), isoleucine (50 mg/mL), and valine (80 mg/mL) compared to the control (dark blue).

We found that when we dissolve leucine, isoleucine, and valine (Figure 1A) at an elevated temperature of 90 °C so as to obtain a homogenous monomeric solution and then cool the solution down gradually, we could observe the self-assembly and formation of ordered structures. Using transmission electron microscopy (TEM), we showed that the BCAAs form ordered and elongated fibril assemblies that resemble the morphology of amyloidal structures (Figure 1B). The structures formed appear in various densities, from confined single fibrils to network-like fibrils (Figure S1). Interestingly, high-resolution scanning electron microscopy (HR-SEM) analysis revealed that leucine, isoleucine, and valine also form large crystal structures (Figure 1C), much larger than the assemblies observed using transmission electron microscopy. The formation of crystals is likely to be a result of the HR-SEM sample preparation procedures in which the drying leads to extremely high concentrations just prior to the complete evaporation of the aqueous solutions. Powder X-ray diffraction (PXRD) analysis confirmed that these crystals possess the same lattice structures as those found in the literature [24,25,26] (Figure S2). Moreover, we were able to monitor the formation of the BCAA crystals, starting from nucleation to polymerization (Figure S3).

### 2.2. Kinetic Analysis of Metabolite Structure Formation

Fibrillar assemblies formed by several metabolites were recently reported to exhibit amyloid-like properties including binding to amyloid-specific dyes [19,20,27,28]. Thus, we decided to test whether the BCAA assemblies display similar characteristics. We used the amyloid-specific fluorescent dyes, Thioflavin-T (ThT) and ProteoStat^®^ (Enzo Life Sciences, Inc., Farmingdale, NY, USA), which bind to β-sheet-rich structures. These reagents change their fluorescence properties upon binding to ordered amyloid-like structures [29,30]. Indeed, when various concentrations of BCAA assemblies were incubated with ProteoStat^®^ dye, a dose-dependent fluorescence signal was obtained (Figure 1D). Moreover, by measuring the ThT fluorescence signal over time, we were able to detect the kinetics of the BCAA structure formation. The metabolite fibrils presented a distinctive time-dependent increase in fluorescence similar to amyloid assemblies [31], while the control solution without metabolites did not (Figure 1E).

### 2.3. Cytotoxicity and Apoptotic Effect of BCAA Assemblies

**Figure 2:**
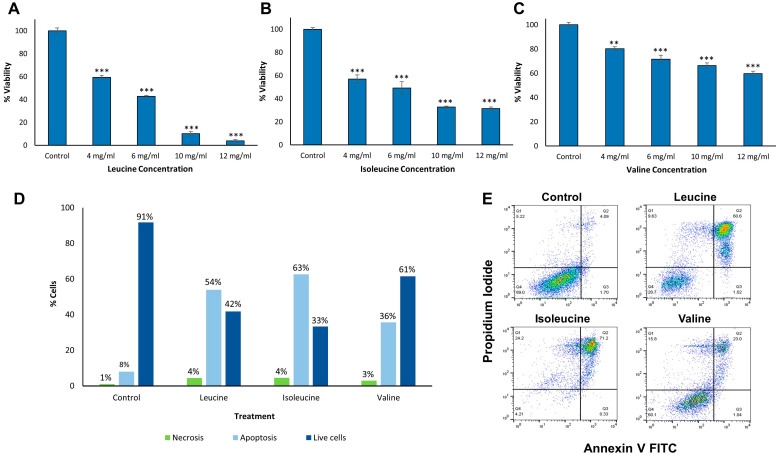
Cytotoxicity and apoptotic activity of BCAA assemblies in neuroblastoma SH-SY5Y cells. Metabolites were dissolved at 90 °C in a cell growth medium followed by the gradual cooling of the solutions. SH-SY5Y cells were incubated in a medium containing the metabolites at the stated concentrations for 48 h; control cells were incubated in a medium without any addition of metabolites. (**A**–**C**) MTT cell viability assay of SH-SY5Y cells treated with assemblies of (**A**) leucine, (**B**) isoleucine, and (**C**) valine. Absorbance was determined at 570 nm and 680 nm. (**D**,**E**) Apoptosis assay of SH-SY5Y cells incubated with 15 mg/mL BCAA structures or media as a control for 48 h. After incubation, annexin V-FITC and PI were added to the cells, followed by flow cytometry analysis using a single laser emitting excitation light at 488 nm. (**D**) Chart presenting the quantification of flow cytometry results. Apoptosis is represented in light blue (early + late apoptosis), necrosis in green, and live cells in dark blue. (**E**) Flow cytometry plots of the annexin V/PI double-staining assay. Q1, annexin V-FITC(-) PI(+)—cells undergoing necrosis; Q2, annexin V-FITC(+) PI(+)—cells in late apoptosis and undergoing secondary necrosis; Q3, annexin V-FITC(+) PI(-)—cells in early apoptosis; Q4, annexin V-FITC(-) PI(-)—live cells. The results represent three biological repeats, error whiskers on bars represent one SD, Student’s *t*-test, ** *p* < 0.01, *** *p* < 0.001 (all statistics are in relation to the control).

Previous studies demonstrated that metabolite amyloid-like structures bear a cytotoxic effect, inducing apoptotic cell death in cultured neuronal cells, similar to the effect of protein and polypeptide amyloids [19,20,32]. We therefore examined the cytotoxicity and specifically the apoptotic activity of BCAA assemblies on cultured human neuroblastoma cells (SH-SY5Y) (Figure 2). We prepared the assemblies by dissolving various concentrations of the BCAAs in a culture medium, followed by the gradual cooling of the solutions. Cells were incubated with media containing the assemblies for 48 h. A medium without the assemblies served as a control. To exclude absorbance changes derived from the metabolite assemblies, background measurements containing the same concentrations of BCAA assemblies in media, in the absence of cells, were performed and subtracted from the cell-based viability data. Using the 3-(4,5-dimethylthiazolyl-2)-2,5- diphenyltetrazolium bromide (MTT) cell viability assay, we tested the toxicity of the assemblies. The assemblies displayed a dose-dependent toxic effect on the neuronal cells, as the viability of the cells decreased gradually in correlation with increasing concentrations of the metabolite structures (Figure 2A–C).

Next, to better understand the mechanism of BCAA assemblies’ cytotoxicity and distinguish between apoptosis and necrosis, an annexin V and propidium iodide (PI) apoptosis assay was used, followed by fluorescence-activated cell sorting (FACS). To allow significant toxicity, high concentrations of BCAA assemblies in cell media were prepared as described above for the MTT assay and cultured SH-SY5Y cells were treated for 48 h. As a control, cells were incubated with media without the metabolites. This assay yielded similar results to those observed in the cytotoxicity assay, demonstrating that apoptosis was the main pathway inducing SH-SY5Y cell death following treatment with the metabolite assemblies (Figure 2D,E). The observation of the differential apoptotic activity of the metabolites as compared to their overall toxicity as reflected by the MTT assay may suggest the combination of the pro-apoptotic and membrane destabilization activity of the metabolite assemblies. Overall, both assays imply that the metabolite assemblies not only exert a cytotoxic effect, similar to the effect of amyloid structures but also induce programmed cell death, which is consistent with the mechanism suggested for protein and polypeptide amyloids [32].

### 2.4. Cellular Detection of Metabolite Assemblies Using Amyloid-Specific Dye

**Figure 3:**
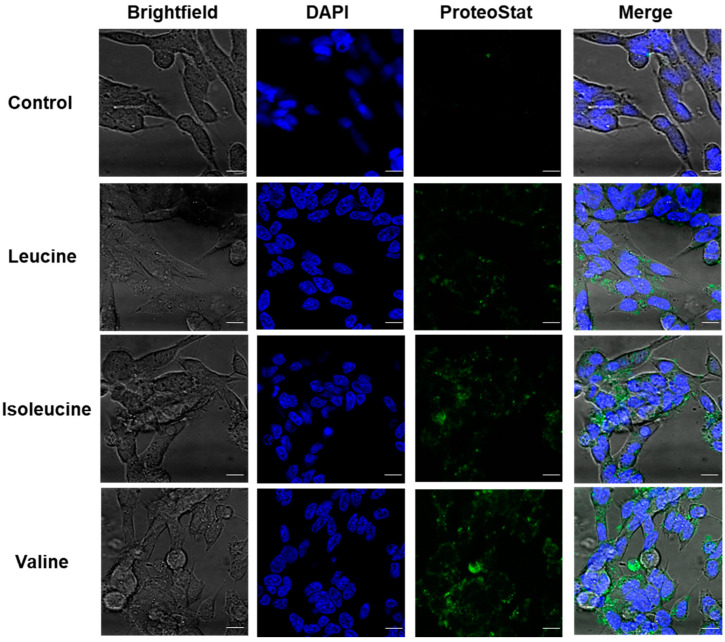
Detection of BCAA amyloid-like structures in treated neuroblastoma cells. Representative confocal images of treated and untreated SH-SY5Y cells using ProteoStat^®^ staining. Cells were treated for 24 h with BCAA assemblies (4 mg/mL) prepared in cell media. The control reflects cells incubated with a medium that was treated in the same manner but without the addition of metabolites. ProteoStat^®^ staining (green), DAPI staining (blue). Scale bars: 10 μm.

Up to this point, we observed the structures formed using dyes in vitro. Next, we sought to detect the metabolite amyloid-like structures in human neuroblastoma SH-SY5Y cells. BCAA assembly solutions (4 mg/mL) were prepared by dissolving the metabolite in cell culture media (as described above for the MTT studies). A medium that was prepared in the same manner but without the addition of metabolites was used as a control. SH-SY5Y cells were incubated with or without the BCAA structures for 24 h. The treated and untreated control cells were then stained with the ProteoStat^®^ amyloid-specific fluorescent dye that was recently shown to be indicative of metabolite amyloid fibrils [17,27]. Finally, the cells were observed using confocal microscopy (Figure 3 and Figure S4), demonstrating the clear staining with amyloid-indicative dye in treated SH-SY5Y cells, while no staining was detected in the untreated cells.

### 2.5. Inhibition of BCAA Self-Assembly by Common Amyloid Fibril Formation Inhibitors

**Figure 4:**
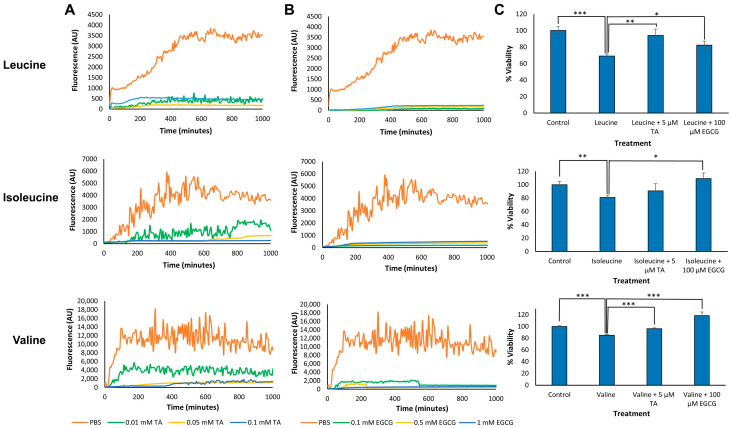
Inhibition of BCAA self-assembly and cytotoxicity by polyphenol-based inhibitors. (**A**,**B**) Leucine (30 mg/mL), isoleucine (40 mg/mL), and valine (80 mg/mL) were dissolved at 90 °C in PBS and mixed with the inhibitors, either (**A**) tannic acid (TA) or (**B**) epigallocatechin gallate (EGCG), at the stated concentrations or with PBS as a control, followed by addition of 40 μM ThT in PBS. ThT emission data at 480 nm (excitation at 450 nm) were measured over time. The results represent three biological repeats. (**C**) BCAA assemblies (10 mg/mL) were prepared in a cell growth medium in the presence of TA (5 µM) or EGCG (100 µM). SH-SY5Y cells were incubated with the metabolites in the absence or presence of the inhibitors, and control cells were incubated with a medium with no metabolites, which was treated in the same manner. After 24 hours of incubation, the MTT reagent was added, and the cell viability was evaluated. Absorbance was determined at 570 nm and 680 nm. The results represent three biological repeats, error whiskers on bars represent one SD, Student’s *t*-test, * *p* < 0.05, ** *p* < 0.01, *** *p* < 0.001.

The formation of amyloid-like fibrils by BCAAs may not only propose a new amyloid-like etiology for MSUD but also suggest that small molecules that are known to inhibit the organization of protein and polypeptide amyloid structures can also affect BCAA self-assembly. The inhibitory potential of polyphenols was demonstrated in the context of various amyloidogenic proteins and polypeptides [33,34,35,36]. Two polyphenols, tannic acid (TA) and epigallocatechin gallate (EGCG), which were previously found to inhibit metabolite amyloid aggregation [17,27,37], were selected to study the mechanism of metabolite self-assembly inhibition. We were interested in examining whether these molecules can inhibit the formation of structures by BCAAs. First, we tested the inhibitory potential of the polyphenols on the BCAA assembly kinetics using the ThT binding assay (Figure 4). This fluorometric technique allows the monitoring of the kinetics of the amyloid fibril assembly process as well as the testing of compounds that have the potential to inhibit their assembly. The ThT fluorescence assay demonstrated that both TA (Figure 4A) and EGCG (Figure 4B), inhibited the formation of assemblies by leucine, isoleucine, and valine, as reflected by the significant reduction in the ThT fluorescence intensity.

Later, we examined the correlation between the inhibition of BCAA assembly formation by the polyphenol inhibitors and the resulting cytotoxicity. For this purpose, TA and EGCG were added to BCAA monomeric solutions prepared in cell growth media. The concentration of the inhibitors was lower than in the in vitro experiments as higher concentrations were toxic to the cells. SH-SY5Y cells were incubated with BCAA solutions for 24 hours in the presence or absence of inhibitors (Figure 4C). The MTT assay indicated that in the presence of EGCG, cell viability increased significantly following treatment. TA also increased the viability of cells incubated with leucine or valine, while cells that were incubated with isoleucine solutions likewise presented a trend of reduced cytotoxicity but not in a significant manner.

## 3. Discussion

While MSUD is considered an orphan disease, it should be noted that, collectively, IEMs are quite frequent with an incidence of about 1 in 2500 births. Accordingly, IEMs represent a significant origin of global child morbidity and mortality, comprising a notable proportion of child deaths currently not defined in the global modeling efforts [38,39]. While MSUD is characterized by the accumulation of leucine, isoleucine, and valine due to mutations in the BCKDH complex, there is a lack of precise information on the mechanism by which the neurological symptoms are caused, thus impeding the development of disease-modifying treatments other than alternative diets [40]. Therefore, there is an essential, unmet need to understand the biological processes that induce the observed MSUD pathology to identify therapeutic targets.

The pathological self-assembly of fibrillar structures was traditionally attributed only to proteins and polypeptides, and their abnormal proteostasis (protein homeostasis) was well characterized and studied [41,42,43,44,45]. By applying a reductionist approach, our group has recently established the field of metabolite self-assembly by expanding the amyloid hypothesis to include amyloid-like structures formed by various metabolites [18,20,46,47,48]. Thus, abnormal metabolostasis (metabolite homeostasis) could also be viewed as central disease etiology [49]. Here, we describe, for the first time, the self-assembly of the BCAAs into ordered fibrillar structures (Figure 1B). Moreover, we have shown the kinetics of the metabolite structure formation using amyloid-specific dyes (Figure 2), which also allowed the cellular detection of the structures (Figure 4). Furthermore, these structures were shown to have a cytotoxic effect that induces apoptosis in neural model cells (Figure 3). These accumulating results presenting the amyloid-like properties of these assemblies confirm BCAA structures as toxicity-causing agents.

The resemblance we demonstrated between the metabolite assemblies, as compared to protein and polypeptide amyloids, allowed us to utilize previously characterized amyloid aggregation inhibitors as key initial leads for potential inhibitors of the metabolite assemblies. One extensively explored group of inhibitors is polyphenol molecules [37,50,51]. In this current study, we used the EGCG and TA polyphenols, two aggregation inhibitors recently reported to inhibit fibril formation by metabolites [17,27,39]. Indeed, EGCG and TA were found to hinder the self-assembly process of the BCAAs, even when applied at a very low molar ratio. Moreover, the formation of the toxic metabolite structures was also reduced following the addition of the inhibitors, as was explored by cell viability analysis performed on neuroblastoma cells (Figure 4). Knowing that the BCAA assemblies’ effects are mitigated by treatment with amyloid inhibitors suggests that targeting BCAA aggregation may be a viable therapeutic strategy for MSUD.

Current therapeutic strategies for MSUD are predominantly dietary management and medical interventions. Ongoing research has explored alternative treatment approaches such as gene therapy [52]. In addition, recent studies have shown that the use of chemical chaperones is efficient in inhibiting metabolite self-assembly [53,54] and, more specifically, can mitigate assembly defects caused by MSUD mutations [55]. However, to date, none of the approaches have led to the initiation of new clinical trials for drug therapies specifically targeting MSUD. Thus, the inhibitory effect of using polyphenols demonstrated in this work could play a pivotal role in the design and development of specific small molecular inhibitors of the formation of BCAA toxic assemblies.

Overall, these results have identified BCAAs as building blocks for the formation of amyloid-like structures, which extend the paradigm-shifting concept, suggesting that the toxicity in IEM disorders may be associated with the formation of these structures. This holds significant implications for both understanding the pathophysiology of MSUD and potentially guiding future therapeutic approaches. Altogether, the findings from this study can pave the way for additional research to be conducted on the development of small molecule inhibitors that effectively disrupt BCAA self-assembly and thus open up new avenues for future therapy for this disease.

## 4. Materials and Methods

### 4.1. Materials

#### 4.1.1. Reagents and Kits

L-leucine, L-isoleucine, L-valine, MTT reagent thiazolyl blue tetrazolium bromide epigallocatechin gallate (EGCG), tannic acid (TA), (Sigma-Aldrich, Burlington, MA, USA), Thioflavin T (ThT) (Biological Industries, Kibbutz Beit Haemek, Israel), ProteoStat^®^ (Enzo Life Sciences, Farmingdale, NY, USA), MEBCYTO apoptosis kit (MBL International, Des Plaines, IL, USA).

#### 4.1.2. Cell Lines

The cell line used in this study was neuroblastoma SH-SY5Y (ATCC^®^ CRL-2266™).

### 4.2. Experimental Methods

#### 4.2.1. Metabolite Structure Formation

Fresh stock solutions of BCAA assemblies were prepared by dissolving the metabolites at 90 °C in phosphate-buffered saline (PBS) (Biological Industries) or in cell culture media at various concentrations to obtain monomeric solutions of the metabolite, followed by the overnight gradual cooling of the solution inside the thermo-shaker until it reached room temperature. Such a technique of heating and gradual cooling was previously used by our group and others to allow the self-assembly of metabolites and ultra-short peptides [18,19,20].

#### 4.2.2. Transmission Electron Microscopy (TEM)

Metabolites were dissolved at 90 °C in PBS at various concentrations followed by the gradual cooling of the solution. On the following day, 10 μL of samples were placed on 400-mesh copper grids. After 2 min, excess fluids were removed. Samples were viewed using a JEOL 1200EX electron microscope operating at 80 kV (JEOL, Peabody, MA, USA).

#### 4.2.3. High-Resolution Scanning Electron Microscopy (HR-SEM)

Following 4 mg/mL BCAA structure formation, 10 µL of each sample was deposited on a glass coverslip and left to dry at room temperature. The slides were then fixed on a stub using carbon tape. Next, the samples were coated with chromium and viewed using a JSM-6700 field-emission high-resolution scanning electron microscope (HR-SEM) (Jeol, Tokyo, Japan), equipped with a cold field emission gun, operating at 10 kV.

#### 4.2.4. ProteoStat^®^ Fluorescence Endpoint Measurements

Metabolites were dissolved at various concentrations at 90 °C in PBS and plated in a 96-well black plate together with ProteoStat^®^ (prepared according to the manufacturer’s instructions) at a 1:100 ratio. Following overnight incubation at room temperature, the ProteoStat^®^ emission signal at 620 nm (excitation at 485 nm) was measured using a Tecan^TM^ Infinite 200 PRO plate reader (Tecan Trading AG, Männedorf, Switzerland). The displayed results are representative of three biological experiments performed in triplicate.

#### 4.2.5. ThT Fluorescence Kinetics Assay

Metabolites were dissolved at 90 °C in PBS at various concentrations and monomeric solutions were obtained. Samples containing polyphenols (EGCG or TA) were immediately mixed with the inhibitors at the stated concentrations. As a control, metabolites were diluted with PBS alone to the same final concentrations. An amount of 40 µM ThT in PBS (final concentration) was added to a black 96-well, clear, and flat-bottom microplate. Next, the solutions were plated, and the self-assembly kinetics were recorded over time. ThT emission data at 480 nm (excitation at 440 nm) were measured using a Tecan™ SPARK 10 M plate reader (Tecan Trading AG, Männedorf, Switzerland). The displayed results are representative of three biological experiments performed in triplicate.

#### 4.2.6. Cytotoxicity Experiments

SH-SY5Y neuroblastoma cells (2 × 10^4^ cells/well) were cultured in DMEM F12 (Ham’s; 1:1) supplemented with 10% fetal bovine serum (FBS) (Biological Industries) in 96-well tissue microplates (100 μL per well) and allowed to adhere overnight at 37 °C. Half of each plate was plated with cells, while the other half later serving as a control containing solutions only. The treatment solutions were prepared as follows: metabolites were dissolved at various concentrations at 90 °C in cell media without FBS, followed by the gradual cooling of the solutions. For solutions containing polyphenols, metabolites were similarly dissolved in cell media and mixed with EGCG or TA (final concentration of 0.1 mM and 5 μM, respectively, stock solution dissolved in cell medium) before gradual cooling. Controls of media alone or of media supplemented with the inhibitors, without metabolites, were examined as well. Cell media were replaced and cells were treated with the solutions (100 μL per well) followed by 24 to 48 h of incubation at 37 °C. Cell viability was evaluated using the 3-(4,5-dimethylthiazolyl-2)-2,5- diphenyltetrazolium bromide (MTT) cell proliferation assay according to the manufacturer’s instructions. Briefly, after overnight incubation at 37 °C with the treatments, 10 µL of 5 mg/mL MTT reagent dissolved in PBS was added to each well, followed by four additional hours of incubation at 37 °C. Next, 100 µL DMSO (ACROS Organics) was added to the wells, followed by 30 min of incubation at 37 °C in the dark. Finally, color intensity was measured using a plate reader at 570 nm and background subtraction at 680 nm. Blank measurements of the solutions without cells were, respectively, subtracted. Values are means ± SD. For inhibitors, a two-tailed Student’s *t*-test was performed with two groups compared. The displayed results are representative of three biological experiments performed in triplicate.

#### 4.2.7. Apoptosis Assay

SH-SY5Y cells (2 × 10^5^ cells/well) were cultured in 24-well plates in DMEM F12 supplemented with 10% FBS and were allowed to adhere overnight at 37 °C. The treatment solutions containing 15 mg/mL of BCAA assemblies were prepared as described above for the MTT assay. Cells were treated with the solutions followed by 48 h of incubation at 37 °C. Control cells were incubated with a medium that was treated in the same manner but without metabolites. The apoptotic effect was evaluated using the MEBCYTO Apoptosis kit according to the manufacturer’s instructions. Briefly, the adherent cells were trypsinized, detached, and combined with floating cells from the incubated growth medium. Cells were then centrifuged and washed once with PBS and once with binding buffer. Cells were subsequently incubated with annexin V–FITC and PI for 15 min in the dark, resuspended in 100 μL of binding buffer, and analyzed by flow cytometry using a single laser-emitting excitation light at 488 nm. Data from 10,000 cells were acquired using BD FACSort and the CellQuest software (version 5.2.1, BD Biosciences). Analysis was performed using the FlowJo software (TreeStar, version 14). The displayed results are representative of three biological experiments performed in triplicate.

#### 4.2.8. Cellular Detection of BCAA Assemblies Using ProteoStat^®^

An amount of 2 × 10^5^ cells/mL was cultured in DMEM F12 supplemented with 10% FBS in a 24-well plate on glass slides coated with 0.01% Poly-L-Lysine. Upon reaching ~70–80% confluency, cells were treated with BCAA assemblies (4 mg/mL dissolved at 90 °C in cell media without FBS, followed by the gradual cooling of the solution). After 24 h of incubation, the cells were immediately supplemented with ProtesoStat dye diluted 1:250 in PBS, followed by incubation for 15 min at room temperature protected from light. Cells were imaged using a Leica TCS SP8 laser confocal microscope with ×63 1.4 NA oil objectives (Leica Microsystems, Wetzlar, Germany). An argon laser with a 488 excitation line was used for ProteoStat^®^ (emission wavelength, 500–600 nm). The results displayed are representative of three biological experiments.

## Data Availability

The data presented in this study are available in the article itself and in the supplementary information.

## Supplementary Materials

The supporting information can be downloaded at: https://www.mdpi.com/1422-0067/24/21/15999/s1.
