# Supplementary material for: Branched-Chain Amino Acid Assembly into Amyloid-like Fibrils Provides a New Paradigm for Maple Syrup Urine Disease Pathology
Source: Int J Mol Sci. 2023 Nov 6;24(21):15999. doi: 10.3390/ijms242115999 (PMC10650742; doi:10.3390/ijms242115999)
Supplement: Supplementary file 1 [file ijms-24-15999-s001.zip › ijms-2554319-supplementary.pdf]

Supplementary information

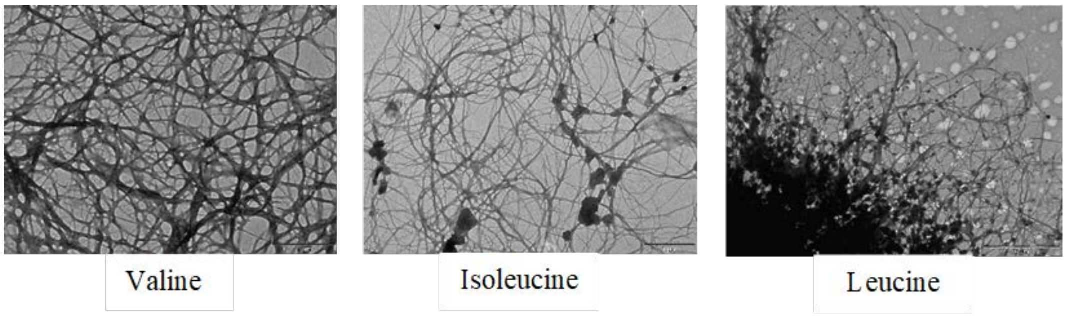

Figure S1. Network-like fibrils of all three metabolites. Transmission electron microscope images showing condensed fibrillar structures of valine, isoleucine and leucine (scale bars: valine and isoleucine - 2 μm, leucine - 1 μm).

<u>Powder X-ray diffraction (PXRD)</u>

4 mg/mL of each of the BCAAs were dissolved at 90 °C in double distilled water (DDW) and allowed to self-assemble by incubating at room temperature for one week. The assembled fibrils were then lyophilized to achieve powders. X-ray diffraction was collected using a Bruker D8 Discover diffractometer with LYNXEYE EX linear position detector.

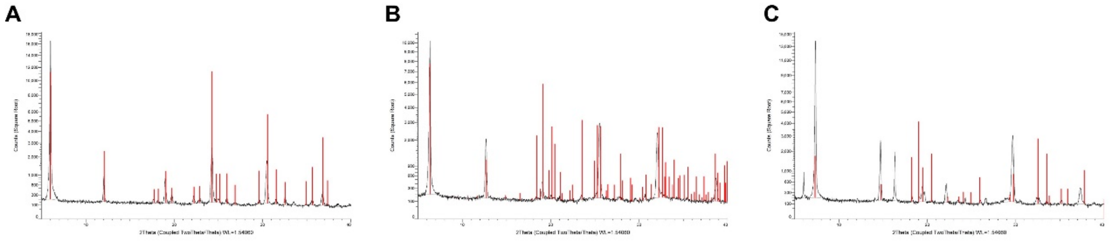

Figure S2. PXRD patterns of BCAA crystals. A-C. Experimental PXRD patterns were collected for BCAA fibrils (black) and compared to the previously published PXRD patterns of the BCAA (red). **A.** Leucine (Powder diffraction file (PDF) 00-030-1779). **B**. Isoleucine (PDF 02-067-6944). **C**. Valine (PDF 00-010-0712)[1–3].

Monitoring of crystal structure formation

We were interested in monitoring the formation of the BCAA crystals, starting from nucleation to polymerization. Leucine (35 mg/mL), isoleucine (45 mg/mL) and valine (90 mg/mL) were dissolved at 90 °C in DDW to allow the complete dissolution of the BCAA to monomers. Once fully dissolved, 400 µl of the hot samples were inserted into a 1 cm path-length quartz cuvette, and then monitored for crystal formation under light microscope. This process was examined under a Nikon Ti-E Inverted microscope, and images were captured by a Zyla scMOS camera at time zero and at fixed-time intervals. We have observed that as early as time zero, nucleation points of crystals started to form. Leucine had the highest crystallization rate, and after 2.5 minutes, the cuvette was filled with crystals. Isoleucine crystals were found to be the smallest with a needle-like morphology, and valine had the lowest number of nucleation events forming relatively large crystals. Notably, the crystals were found to polymerize and elongate mainly in one dimension.

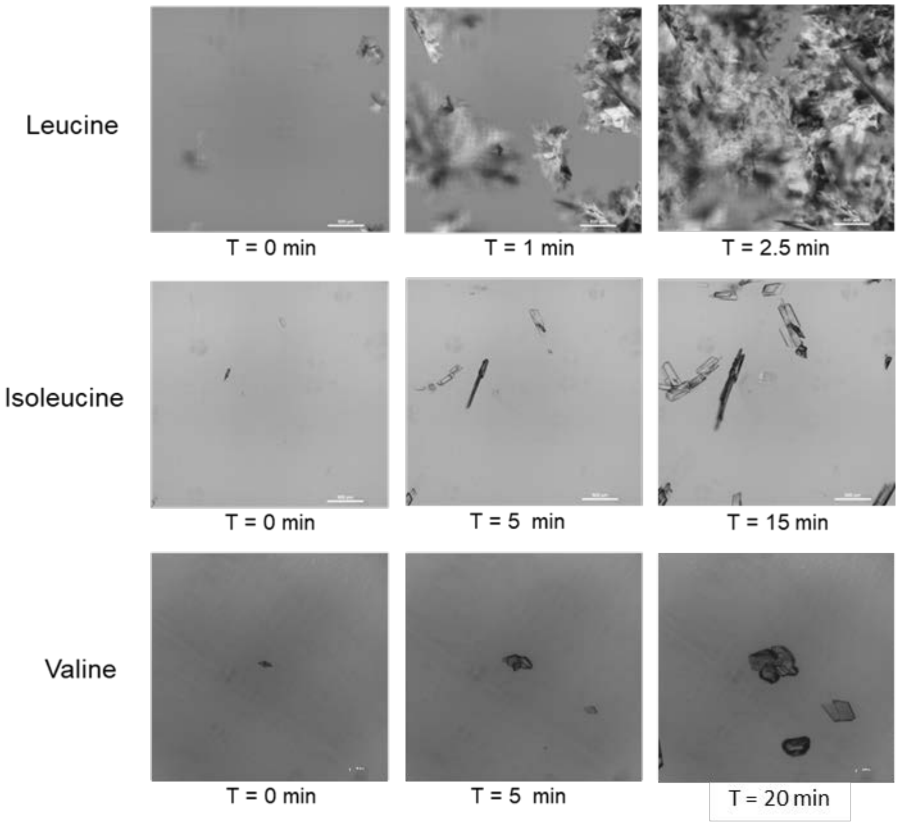

Figure S3. BCAA crystals formation. All metabolites were dissolved at elevated temperature of 90 °C in DDW and were immediately inserted into a quartz cuvette and then time point images were recorded using light microscope. Leucine (35 mg/mL), isoleucine (45 mg/mL) and valine (90 mg/mL). Scale bars: 500 µm.

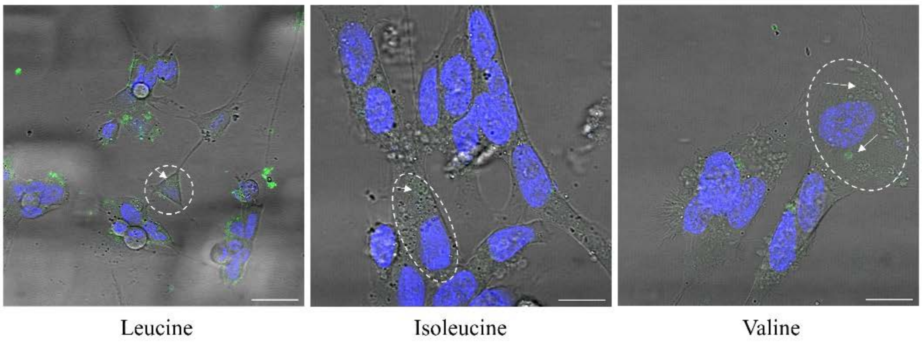

Figure S4. Detection of intracellular BCAA assemblies in neuroblastoma treated cells. Representative confocal images of SH-SY5Y neuroblastoma cells that were treated with BCAA assemblies and stained using the amyloid-specific dye ProteoStat®. BCAA assemblies (green), nucleus (blue). The dashed circles focus on a single cell and the arrows point at intracellular BCAA aggregates. Scale bars: leucine - 25 μm, isoleucine and valine - 10 μm.

References

1. Torii, K. & Iitaka, Y. The crystal structure of L-isoleucine. *Acta Crystallogr. Sect. B Struct. Crystallogr. Cryst. Chem.* (1971) doi:10.1107/s0567740871005612.
2. Torii, K. & Iitaka, Y. The crystal structure of L-valine. *Acta Crystallogr. B.* (1970) doi:10.1107/S0567740870004065.
3. Coll, M., Solans, X., Font-Altaba, M. & Subirana, J. A. Structure of L-leucine: a redetermination. *Acta Crystallogr. Sect. C Cryst. Struct. Commun.* (1986) doi:10.1107/s0108270186095240.
